# LRRC8/VRAC anion channels are required for late stages of spermatid development in mice

**DOI:** 10.1074/jbc.RA118.003853

**Published:** 2018-06-07

**Authors:** Jennifer C. Lück, Dmytro Puchkov, Florian Ullrich, Thomas J. Jentsch

**Affiliations:** From the ‡ Leibniz-Forschungsinstitut für Molekulare Pharmakologie (FMP), D-13125 Berlin, Germany,; the § Max-Delbrück-Centrum für Molekulare Medizin (MDC), D-13125 Berlin, Germany,; the ¶ Graduate Program of the Freie Universität Berlin, 14195 Berlin, Germany, and; the ‖ Neurocure Cluster of Excellence, Charité Universitätsmedizin, 10117 Berlin, Germany

**Keywords:** spermatozoa, spermatogenesis, ion channel, mitochondria, membrane protein, plasma membrane, mouse, ebo, ICl, swell, ICl, vol, VSOR, swelling-activated chloride channel, Cl-channel

## Abstract

Spermatogenesis is a highly complex developmental process that occurs primarily in seminiferous tubules of the testes and requires additional maturation steps in the epididymis and beyond. Mutations in many different genes can lead to defective spermatozoa and hence to male infertility. Some of these genes encode for ion channels and transporters that play roles in various processes such as cellular ion homeostasis, signal transduction, sperm motility, and the acrosome reaction. Here we show that germ cell–specific, but not Sertoli cell–specific, disruption of *Lrrc8a* leads to abnormal sperm and male infertility in mice. LRRC8A (leucine-rich repeat containing 8A) is the only obligatory subunit of heteromeric volume-regulated anion channels (VRACs). Its ablation severely compromises cell volume regulation by completely abolishing the transport of anions and osmolytes through VRACs. Consistent with impaired volume regulation, the cytoplasm of late spermatids appeared swollen. These cells failed to properly reduce their cytoplasm during further development into spermatozoa and later displayed severely disorganized mitochondrial sheaths in the midpiece region, as well as angulated or coiled flagella. These changes, which progressed in severity on the way to the epididymis, resulted in dramatically reduced sperm motility. Our work shows that VRAC, probably through its role in cell volume regulation, is required in a cell-autonomous manner for proper sperm development and explains the male infertility of *Lrrc8a*^−/−^ mice and the spontaneous mouse mutant *ébouriffé*.

## Introduction

Spermatogenesis, the production of male gametes, takes place in seminiferous tubules that harbor Sertoli cells and germ cells at various developmental stages. Sertoli cells envelop and provide a specialized environment for the developing spermatozoa. Following cycles of mitosis and meiosis, spherical, haploid spermatids transform into elongated and polarized spermatozoa (1). This transformation, known as spermiogenesis, includes formation of the acrosome, chromatin condensation, and reshaping of the nucleus that results in the typical sperm head shape and the assembly of a mitochondrial sheath around the axoneme of the flagellum. It culminates in spermiation, in which residual cytoplasm of the elongated spermatids is shed off and spermatozoa are released into the lumen of the seminiferous tubule (2, 3). Seminiferous tubules merge into the rete testis and finally into the efferent ducts (4), which guide spermatozoa to the epididymis. During epididymal transit, spermatozoa undergo further maturation, thereby acquiring abilities required for fertilization of the oocyte (5–7). Mature spermatozoa are then stored in the cauda epididymis until ejaculation.

During their development and maturation, male germ cells are exposed to changes in extracellular osmolality. From roughly isoosmotic conditions in seminiferous tubules, the osmolality increases to up to ∼410 mOsm in the epididymis (5, 7, 8). Although the maintenance of a near-constant cell volume in face of hyper- or hypotonic challenges is crucial for cells in general, this is believed to be of particular importance for male germ cells (7). To counteract shrinkage or swelling under hyper- and hypotonic conditions, cells have developed two mechanisms, namely regulatory volume increase and regulatory volume decrease (RVD),^2^ respectively (9–11). Previous studies demonstrated that the high osmolality in the epididymis is important for sperm maturation (8) and that the ability of spermatozoa to regulate their volume during epididymal transit (5, 12, 13) and in the female reproductive tract (7, 14) can have an effect on their motility. Upon excessive swelling, *e.g.* caused by impaired RVD, spermatozoa change the shape of their flagella to reduce membrane tension (7). This usually results in a coiling or angulation of flagella that impairs their forward motility and thus the ability to pass the female reproductive tract and fertilize the egg (7). Abnormalities of sperm flagella, referred to as teratozoospermia, are a common cause of infertility in mice and men (15–18).

A key player in RVD is the volume-regulated anion channel (VRAC; Ref. 11) (also known as volume-sensitive outwardly rectifying anion channel, or VSOR (19)). These plasma membrane channels, which are ubiquitously expressed in vertebrate cells, are normally closed under resting conditions and open upon cell swelling. VRAC-mediated efflux of organic osmolytes and Cl^−^, the latter paralleled by K^+^ efflux through independent K^+^ channels, decreases intracellular osmolality and thereby reduces cell volume by driving water out of the cell (11, 20). Only recently, VRAC was discovered to be constituted by LRRC8 heteromers (21) that are formed by the obligatory subunit LRRC8A (21, 22) and at least one other member of the LRRC8 protein family (LRRC8B–E) (21). LRRC8 proteins have four transmembrane helices followed by a long cytoplasmic tail that contains many leucine-rich repeats. In part based on their similarity to pannexins and connexins, LRRC8 proteins were believed to assemble to hexameric channels (21, 23, 24), as recently confirmed by cryo-EM structures (25). Depending on the LRRC8 subunit composition, VRACs can also conduct a wide range of organic compounds (26, 27).

The general importance of LRRC8 channels became evident from the severe phenotypes of *Lrrc8a*^−/−^ mice, which were reported shortly after the identification of LRRC8A as an obligatory VRAC constituent (28). These mice display high pre- and postnatal mortality, growth retardation, curly hair, and abnormalities in several tissues. Importantly, females and males lacking LRRC8A are sterile (28) for so-far unknown reasons. It was only recently discovered that the spontaneous mouse mutant *ébouriffé* (29) carries a mutation that truncates the cytoplasmic carboxyl terminus of LRRC8A (30). This mouse mutant shares several pathological features (29) with *Lrrc8a*^−/−^ mice (28). The more benign phenotype of *ébouriffé* mice may be explained by the observation that their VRAC currents are strongly reduced but not abolished (30). The first characterization of *ébouriffé* mice focused on their male sterility, which was attributed to structural defects of sperm cells (29). It remains, however, unclear whether a complete loss of LRRC8A would have similar consequences and whether these pathologies are cell-autonomous outcomes of a reduction of VRAC currents in germ cells or in Sertoli cells.

In this study, we investigated the role of LRRC8A in spermatogenesis using several mouse models. Whereas mice lacking LRRC8A specifically in Sertoli cells were completely fertile, LRRC8A was indispensable in germ cells for the normal development of mature spermatozoa and for male fertility. In the absence of LRRC8A, late spermatids displayed severe disorganization of the mitochondrial sheath in the midpiece region and a drastically swollen cytosolic compartment. Spermatozoa showed flagellar coiling or angulation, features that were previously described with abnormal cell swelling upon RVD failure (7).

## Results

### Differential expression of VRAC forming LRRC8 proteins in the male reproductive system

**Figure 1.**
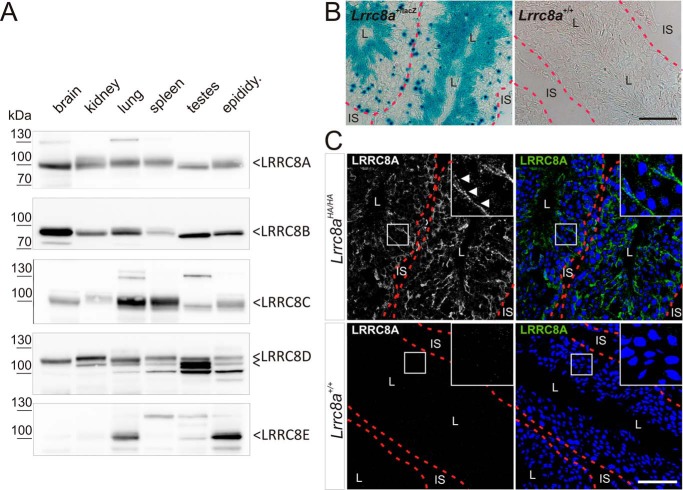
**Expression analysis of LRRC8A and the other VRAC subunits (LRRC8B–E) in the male reproductive system.** *A*, Western blotting analysis of all VRAC subunits (LRRC8A–LRRC8E) in different organs. Specific bands, as determined with appropriate *knock-out* controls, are indicated. *B*, X-Gal staining of sections of *Lrrc8a*^+/LacZ^ and WT (negative control) testis, showing *Lrrc8a* expression in *blue*. Seminiferous tubules are surrounded by a *red dotted line. C*, immunofluorescent labeling of LRRC8A (*green*) in seminiferous tubules of testes (surrounded by *red dotted line*) from *Lrrc8a*^HA/HA^ *knock-in* and control WT mice with anti-HA antibody. *Magnified regions* show a Sertoli cell–characteristic staining pattern (*white arrowheads*). The nuclei stained with DAPI (*blue*). *L*, lumen of seminiferous tubules; *IS*, interstitial space. *Scale bars*, 50 μm (*B* and *C*).

As the basis for exploring the role of VRAC in male fertility, we first determined the expression of all LRRC8 subunits in testis and epididymis. It is generally believed that VRAC is ubiquitously expressed in all vertebrate tissues and cells (11, 20, 31), which is consistent with the wide expression pattern of all LRRC8 genes gleaned from EST database analysis (21). Indeed, Western blotting analysis identified the obligatory VRAC subunit LRRC8A in testis and epididymis and in all other tissues examined (Fig. 1*A*). With the exception of LRRC8E, the other LRRC8 isoforms were also significantly expressed in those tissues (Fig. 1*A*). The glutamate transport-enhancing subunit LRRC8E (26), known to be almost absent from brain and blood cells (21), was prominently expressed in the epididymis but was only barely detectable in testes (Fig. 1*A*).

The testicular expression pattern of *Lrrc8a* was investigated using *knock-in* (KI) mice expressing β-gal under the control of the endogenous *Lrrc8a* promoter (32). Blue LacZ staining was scattered over the whole width of seminiferous tubules (Fig. 1*B*), suggesting that *Lrrc8a* is expressed in Sertoli cells and in germ cells of all developmental stages.

Because the antibodies we have generated against the essential VRAC subunit LRRC8A (21, 27) work only in Western blots (Fig. 1*A*) and not in immunohistochemistry, we generated KI mice in which we fused three hemagglutinin (HA) peptide tags to the carboxyl terminus of LRRC8A. These tags were inserted by CRISPR-Cas9–mediated recombination in fertilized mouse oocytes obtained from crosses of WT and *Lrrc8a*^lox/lox^ (32) mice. The resulting *Lrrc8a*^HA/HA^ and *Lrrc8a*^lox-HA/lox-HA^ mice allowed the detection of LRRC8A by Western blotting and immunohistochemistry using commercial antibodies against the HA tag, with WT mice serving as negative controls. *Lrrc8a*^lox-HA/lox-HA^ mice additionally permit to ascertain Cre-mediated, cell type–specific disruption of *Lrrc8a*.

Consistent with the lacZ staining (Fig. 1*B*), immunofluorescent labeling of testis sections of *Lrrc8a*^HA/HA^ mice revealed broad expression of LRRC8A all over the seminiferous tubules (Fig. 1*C*). We observed a remarkably strong radial staining that extended from the outer circumference to the lumen of the seminiferous tubules, a pattern that is suggestive of Sertoli cells. It is possible that the remaining scattered and weaker labeling represents germ cells, but the low signal intensity precluded a definite conclusion.

### Sertoli cell-specific disruption of LRRC8A does not impair male fertility

**Figure 2.**
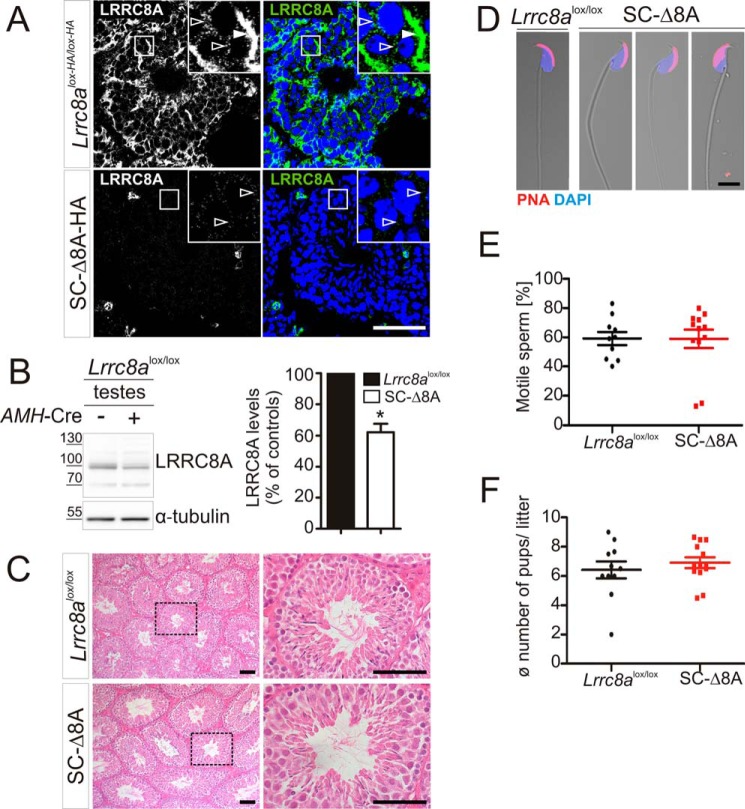
**LRRC8A is dispensable in Sertoli cells for normal spermatogenesis and male fertility.** *A*, immunofluorescent labeling of LRRC8A-3xHA (*green*) in testes of *Lrrc8a*^HA-lox/HA-lox^ and SC-Δ8A-HA mice using an anti-HA antibody. *Magnified regions* show Sertoli cell–characteristic staining pattern (*white arrowhead*) and labeling around germ cell nuclei (*open arrowheads*). *B*, *left panel*, Western blotting analysis of LRRC8A expression in testes of *Lrrc8a*^lox/lox^ mice lacking Cre expression (−) or expressing the Cre recombinase specifically in Sertoli cells (+). *Right panel*, quantification of LRRC8A expression in KO animals compared with *Lrrc8a*^lox/lox^ controls (*n* = 3 independent experiments). *Error bars*, mean ± S.D. *, *p* < 0.05 (paired Student's *t* test). *B* and *C*, H&E staining of paraffin sections of testes from 8-month-old *Lrrc8a*^lox/lox^ control and SC-Δ8A mice. No morphological differences between control and KO mice could be detected. *Dotted areas* (*left panels*) are shown at higher magnification in the *right panels. D*, fluorescent labeling of mature spermatozoa, isolated from cauda epididymides of *Lrrc8a*^lox/lox^ control and SC-Δ8A mice. Acrosomal cap labeled with PNA (*red*) and nuclei with DAPI (*blue*). No abnormalities of SC-Δ8A cells were observed. *E,* unchanged motility of cauda epididymal spermatozoa from SC-Δ8A mice compared with control (*Lrrc8a*^lox/lox^) (*Lrrc8a*^lox/lox^: 59.1 ± 14.13% motile sperm; SC-Δ8A: 58.83 ± 22.09% motile sperm; 10–12 mice per genotype; *p* > 0.05, Mann–Whitney *U* test). *F*, breeding performance of *Lrrc8a*^lox/lox^ control and SC-Δ8A mice, determined by number of pups per litter when mated with Bl6 or *Lrrc8a*^lox/lox^ females (*Lrrc8a*^lox/lox^: 6.4 ± 1.9 pups/litter; SC-Δ8A: 6.9 ± 1.3 pups/litter; 11–13 mice per genotype, *p* > 0.05, Mann–Whitney *U* test). *Scale bars*, 50 μm (*A* and *C*) and 5 μm (*D*).

Considering the complexity of spermatogenesis, including the important interplay of germ cells with Sertoli cells, the male infertility of *Lrrc8a*^−/−^ mice (28) might be due to primary defects in different testicular cell types. For instance, the male infertility of mice lacking the ClC-2 Cl^−^ channel has been tentatively attributed to a defect in Sertoli rather than germ cells (33). To identify the cell type in which absence of LRRC8A causes male infertility, we generated different conditional LRRC8A *knock-out* (KO) mouse models. We first crossed *Lrrc8a*^lox/lox^ (32) or *Lrrc8a*^lox-HA/lox-HA^ mice with AMH-Cre mice (34), which express the Cre-recombinase specifically in Sertoli cells. In the following, we refer to the resulting Sertoli cell-specific LRRC8A KO as SC-Δ8A and SC-Δ8A-HA, respectively. Immunofluorescent analysis of testes from SC-Δ8A-HA mice (Fig. 2*A*) showed that LRRC8A could no longer be detected in seminiferous tubules. However, Western blotting analysis showed only a moderate reduction of LRRC8A protein levels in testes compared with *Lrrc8a*^lox/lox^ controls (Fig. 2*B*), suggesting that LRRC8A is also expressed in testicular cell types other than Sertoli cells. Despite the prominent expression of LRRC8A in Sertoli cells, we failed to detect any morphological changes of SC-Δ8A testes compared with *Lrrc8a*^lox/lox^ controls (Fig. 2*C*). Importantly, spermatozoa isolated from the cauda epididymis of SC-Δ8A mice displayed normal morphology (Fig. 2*D*) and motility (Fig. 2*E*). The breeding efficiency of SC-Δ8A males was also unaffected (Fig. 2*F*). Hence LRRC8A, and by extension VRAC, is dispensable for Sertoli cell function and the development of spermatozoa.

### GC-Δ8A mice show structural abnormalities of sperm flagella and are infertile

**Figure 3.**
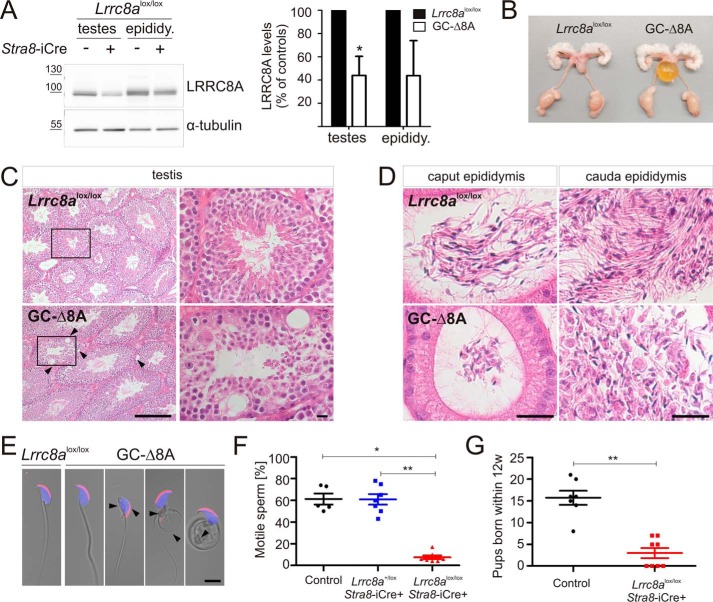
**Germ cell–specific disruption of *Lrrc8a* entails morphological abnormalities and infertility.** *A*, *left panel*, Western blotting analysis of LRRC8A expression in testes and epididymides of *Lrrc8a*^lox/lox^ mice lacking Cre expression (−) or expressing the recombinase specifically in male germ cells (+). *Right panel*, quantification of LRRC8A expression compared with *Lrrc8a*^lox/lox^ controls. The results are from three independent Western blots. *Error bars*, mean ± S.D. *, *p* < 0.05 (paired Student's *t* test). *B*, macroscopic morphology of the male reproductive system of *Lrrc8a*^lox/lox^ control and GC-Δ8A mice. *C*, H&E staining of paraffin sections of testes from 6-month-old *Lrrc8a*^lox/lox^ control and GC-Δ8A mice. Morphology was mostly unchanged, but some GC-Δ8A tubules showed vacuoles (*arrowheads*) and appeared less organized than tubules from control mice (higher magnification of *boxed areas* in *left panels* shown in the *right panels*). *D*, H&E staining of paraffin sections on epididymides from 24-week-old *Lrrc8a*^lox/lox^ control and GC-Δ8A mice. Spermatozoa in caput and cauda epididymis of *Lrrc8a*^lox/lox^ control mice showed straight tails. In GC-Δ8A mice, spermatozoa appeared more clustered and lacked straight tails. *E*, fluorescent labeling of mature spermatozoa, isolated from the cauda epididymides of *Lrrc8a*^lox/lox^ control and GC-Δ8A mice. Acrosomal caps labeled with PNA (*red*) and nuclei with DAPI (*blue*). GC-Δ8A spermatozoa showed multiple morphological abnormalities: bend heads, midpiece disorganization and coiled tails (highlighted with *black arrowheads*). *F*, drastically reduced motility of spermatozoa isolated from cauda epididymis of GC-Δ8A mice compared with heterozygous GC-Δ8A or control (*Lrrc8a*^+/lox^ or *Lrrc8a*^lox/lox^) mice (control, 61.2 ± 11.39% motile sperm; *Stra8*-iCre, *Lrrc8a*^+/lox^, 60.86 ± 12.8% motile sperm; *Stra8*-iCre, *Lrrc8a*^lox/lox^, 7.43 ± 4.69% motile sperm; 5–7 mice per genotype; *, *p* ≤ 0.05; **, *p* ≤ 0.01; one-way analysis of variance with Kruskal–Wallis test). *G*, test breeding performance of control (*Lrrc8a*^+/lox^ or *Lrrc8a*^lox/lox^) and GC-Δ8A (*Stra8*-iCre; *Lrrc8a*^lox/lox^) mice when mated with Bl6 or *Lrrc8a*^lox/lox^ females as determined by the number of pups produced over a period of 12 weeks (control: 3 ± 3.3 pups/12 weeks; GC-Δ8A: 15.7 ± 4.3 pups/12 weeks; 7–10 male mice/genotype; **, *p* ≤ 0.01; Mann–Whitney *U* test). *Scale bars*, 50 μm (*C*), 25 μm (*D*), and 5 μm (*E*).

We crossed *Lrrc8a*^lox/lox^ or *Lrrc8a*^lox-HA/lox-HA^ mice with Stra8-iCre mice (35) to obtain mice specifically lacking LRRC8A in germ cells (in the following referred to as GC-Δ8A mice). The specificity of Cre expression had been previously established using reporter mice (35, 36). LRRC8A protein levels were drastically reduced in GC-Δ8A testes (Fig. 3*A*), indicating that LRRC8A is strongly expressed in male germ cells. A modest reduction of LRRC8A protein levels was also observed in epididymis from GC-Δ8A mice (Fig. 3*A*), suggesting that LRRC8A is also expressed in the latest developmental stage of spermatozoa. The male reproductive system of GC-Δ8A mice appeared macroscopically normal (Fig. 3*B*) and, in contrast to the report on *ébouriffé* mice (29), degenerated or vacuolated seminiferous tubules were observed only occasionally in GC-Δ8A testes (Fig. 3*C*). Morphological analysis of the caput and cauda epididymis of GC-Δ8A mice revealed abnormally shaped spermatozoa that lacked straight flagella (Fig. 3*D*). Analysis of spermatozoa from the cauda epididymis attributed the abnormal shape to the presence of coiled tails, disorganization of the midpiece region, and head bending (Fig. 3*E*). We occasionally observed also normal spermatozoa, which can be explained by the known ∼95% deletion efficiency of Stra8-iCre mice (35) (Fig. 3*E*). Heterozygous GC-Δ8A spermatozoa appeared normal (data not shown). As expected from these malformations, the motility of homozygous but not of heterozygous GC-Δ8A spermatozoa was drastically reduced (Fig. 3*F*). Despite regularly observed vaginal plugs, indicating normal mating behavior, libido, copulation, and ejaculation, the breeding performance of GC-Δ8A males was dramatically reduced (Fig. 3*G*).

### Severe disorganization of the midpiece region of GC-Δ8A spermatozoa

**Figure 4.**
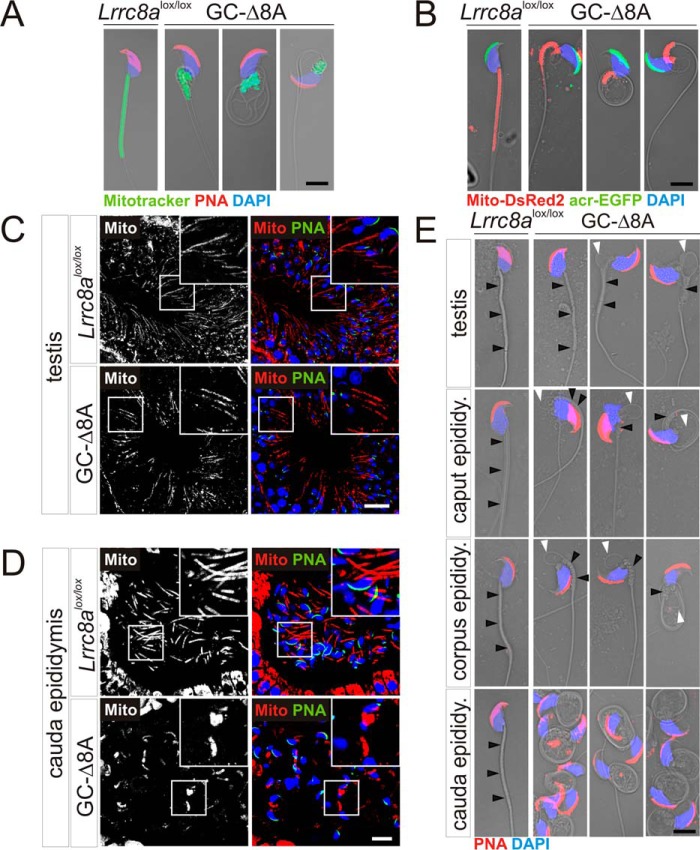
**Progression of flagellum abnormalities in germ cell–specific *Lrrc8a*^−/−^ mice.** *A*, fluorescent labeling of *Lrrc8a*^lox/lox^ control and GC-Δ8A spermatozoa isolated from cauda epididymis. Acrosomal cap labeled with PNA (*red*), the mitochondrial sheath with MitoTracker® (*green*) and nuclei with DAPI (*blue*). *B*, green spermatozoa (expressing EGFP-tagged acrosin and DsRed2-tagged mitochondria) isolated from cauda epididymis of *Lrrc8a*^lox/lox^ control and GC-Δ8A green sperm mice. Sperm were fixed and stained with anti-GFP (*green*) and anti-DsRed (*red*) antibodies. DAPI reveals nuclei (*blue*). *C*, immunohistochemical analysis of testes from *Lrrc8a*^lox/lox^ control and GC-Δ8A green sperm mice. *Magnified regions* show elongated mitochondrial sheaths of sperm in both genotypes. *D*, immunohistochemical analysis of cauda epididymis from *Lrrc8a*^lox/lox^ control and GC-Δ8A green sperm mice. *Magnified regions* show elongated mitochondrial sheaths in *Lrrc8a*^lox/lox^ control mice and mitochondria forming a compact mass in GC-Δ8A mice. *E*, fluorescent labeling of spermatozoa isolated from testes and different epididymal compartments of *Lrrc8a*^lox/lox^ control and GC-Δ8A mice, showing progression of flagellar abnormalities. Mitochondrial sheaths indicated by *black arrowheads. White arrowheads* highlight excess cytoplasm. Acrosomal cap labeled with PNA (*red*) and nuclei with DAPI (*blue*). *Scale bars*, 5 μm (*A*, *B*, and *E*), 10 μm (*D*), and 20 μm (*C*). *Mito*, mitochondria.

Flagellar malformation of spermatozoa is often associated with a disorganization of mitochondria (18, 29, 37–39). MitoTracker® green labeling revealed that mitochondria were evenly distributed along the midpiece region of control, but not of GC-Δ8A spermatozoa (Fig. 4*A*). The mitochondria in GC-Δ8A spermatozoa rather formed a compact mass close to the head like in *ébouriffé* mice (29). We ascertained this finding by crosses with “green sperm” mice (40), which express EGFP in the acrosome and DsRed2 in mitochondria (Fig. 4*B*). In testes from green sperm mice, the majority of GC-Δ8A spermatids displayed an organized elongated, WT-like mitochondrial sheath (Fig. 4*C*). In contrast, GC-Δ8A spermatozoa in the cauda epididymis showed an increased proportion of compacted mitochondria as also observed in isolated spermatozoa (Fig. 4, *A*, *B*, and *D*), suggesting a gradual development of the malformation along the way from the testes to the epididymis. This observation was further corroborated by analysis of isolated spermatozoa. When isolated from GC-Δ8A testes, a large fraction of spermatozoa appeared normal, whereas the remainder showed an enlarged cytoplasm with either normal or malformed midpiece region (Fig. 4*E*). In contrast, almost all spermatozoa isolated from the epididymis were abnormal. In the most proximal part of the epididymis, the caput, we found spermatozoa with different degrees of malformation such as enlarged cytoplasm with normally organized or clustered mitochondria. By contrast, all malformed spermatozoa from the corpus and cauda epididymis displayed disorganized mitochondrial sheaths. In addition, spermatozoa from the corpus region occasionally and from the caudal region almost always showed flagellar coiling (Fig. 4*E*). These observations strongly suggest that the malformation started upon the release of spermatozoa from Sertoli cells and progressed during the transport to the epididymis and then throughout epididymal transit.

### VRAC loss results in gradual swelling of late-stage spermatids

**Figure 5.**
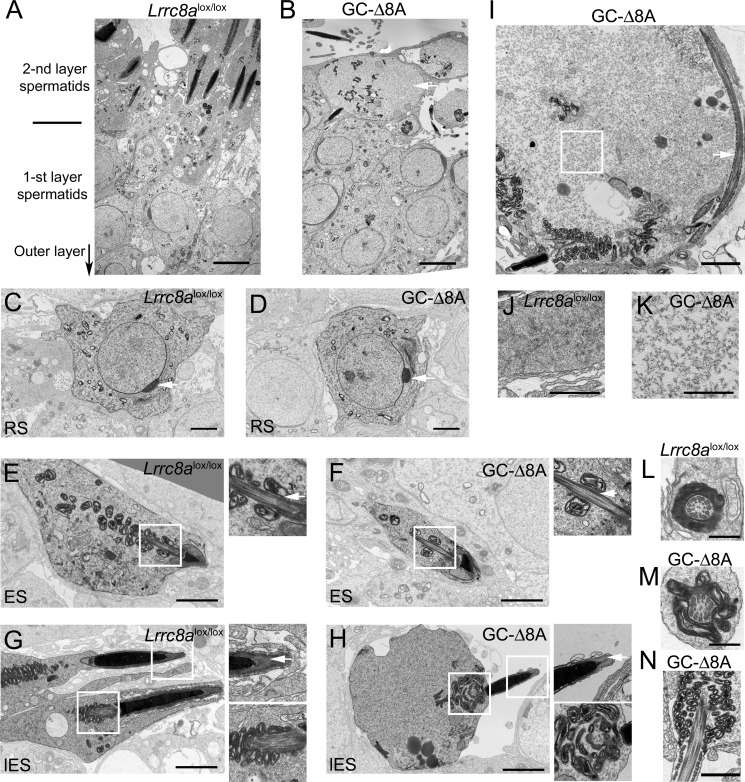
**VRAC loss results in swelling of late stage spermatids in testis.** *A* and *B*, TEM images of stage-matched seminiferous tubules of *Lrrc8a*^lox/lox^ control and GC-Δ8A mice. The *arrow* points in the direction of the outer layer of seminiferous tubules, which harbors spermatogonia and spermatocytes (outside the field of view). The first spermatid layer contains spermatids from round Golgi phase to the end of acrosome phase; the second layer contains maturing spermatids up to spermiation into the lumen. Note in *B* the huge cytoplasm of one of the spermatids from the second layer close to the lumen (*white arrow*). *Scale bars*, 5 μm (*A* and *B*). *C* and *D*, round spermatids (*RS*) from the first layer from *Lrrc8a*^lox/lox^ control and GC-Δ8A mice. No difference was detected between genotypes. Note the properly formed acrosome of a cap phase in both genotypes (*arrows*). *Scale bars*, 2 μm (*C* and *D*). *E* and *F*, earlier phase elongated spermatids (*ES*). There is no difference between genotypes. Mitochondria begin to attach to axonemes (*arrows* in *magnified regions*). *Scale bars*, 2 μm (1 μm for the zoom) (*E* and *F*). *G*, late-phase elongated spermatid (*lES*) close to lumen from control testis. *H*, late phase elongated spermatid at the lumen/second spermatid layer interface from GC-Δ8A mice. Note properly condensed chromatin and acrosomal cap (*arrow in magnified region*), but swollen round cytoplasm and multilayered mitochondria accumulation close to the nucleus. *Scale bars*, 2 μm (1 μm for zoom) (*G* and *H*). *I*, severely affected late phase elongated spermatid at the lumen/second spermatid layer interface from GC-Δ8A mice with swollen cytoplasm, coiled axoneme (*arrow*) and disorganized mitochondria. *Scale bar*, 2 μm (*I*). *J*, *magnified region* of cytoplasm from *Lrrc8a*^lox/lox^ control late elongated spermatid. *K*, *magnified region* of cytoplasm from GC-Δ8A late elongated spermatid (*box* from *I*). Note that cytoplasm is lighter and ribosomes are father apart. *Scale bars*, 1 μm (*J* and *K*). *L*, cross-section through midpiece of a *Lrrc8a*^lox/lox^ control late spermatid/spermatozoon. Note the tight mitochondrial sheath. *M*, cross-section through a midpiece of a GC-Δ8A late spermatid/spermatozoon. Note the moderately disturbed mitochondrial sheath. *Scale bars*, 500 nm (*L* and *M*). *N*, longitude section through the midpiece of a GC-Δ8A late spermatid/spermatozoon. Note the heavily disorganized mitochondrial sheath. *Scale bar*, 1 μm (*N*).

In accord with the gradual appearance of morphological aberrations during spermatogenesis, transmission EM (TEM) analysis revealed no differences between the genotypes in the first, most peripheral layer of spermatids composed of so-called round spermatids (Fig. 5, *A–D*). No cytoplasmic swelling, changes in cytoplasm electron density, or failures in acrosome formation were detected at these early phases of spermatid development (Fig. 5, *C* and *D*). The subsequent early stages of elongated spermatids also appear normal, with mitochondria gathering and adhering around the axoneme (Fig. 5, *E* and *F*). No significant abnormalities could be detected in chromatin condensation and the formation of typically shaped nuclei below acrosomal caps up to the very late elongated spermatid phases (Fig. 5, *G* and *H*).

Abnormal cells could only be detected in the innermost part of GC-Δ8A seminiferous tubules, which harbors the second spermatid layer, where cells are in direct contact with the tubular lumen (Fig. 5, *B*, *H*, and *I*). At this location, GC-Δ8A spermatids frequently had round, swollen cytoplasm directly adjacent to correctly formed nuclei and acrosomal caps (Fig. 5, *B*, *H*, and *I*). By contrast, at this phase of development, spermatids from controls have much less and rather elongated cytoplasm and a tightly packed mitochondrial sheath around correctly formed axonemes (Fig. 5*G*).

The diameter of lumen-adjacent GC-Δ8A spermatids, when measured along a line parallel to the lumen/cell layer interface, was up to 18 μm, compared with 6–8 μm in elongated spermatids from control mice. Lighter cytoplasm and more space between individual ribosomes in late-phase GC-Δ8A spermatids as compared with control (Fig. 5, *J* and *K*) suggested that cytoplasmic swelling and not just a failure to proceed with the abscission of the residual cytoplasm is a cause for aberrant morphology of late spermatids. Mitochondria of GC-Δ8A spermatids start to migrate and cluster (Fig. 5, *H*, *I*, and *N*). Mitochondrial sheath disorganization is likely a gradual process because one can find parallel and cross-sections through the midpiece region of elongated GC-Δ8A spermatids containing relatively intact mitochondrial sheath enveloping axonemes (Fig. 5*M*), although they are less compact compared with control spermatids (Fig. 5*L*). Excess of swollen residual cytoplasm in GC-Δ8A spermatids likely promotes redistribution of spermatid mitochondria into the swollen cytoplasmic compartment and mitochondrial clustering because it is often observed in mutants that fail to properly form sperm heads or to eliminate residual cytoplasm (36, 39, 41). Flagellar coiling and midpiece disorganization described for those mutant models of globozoospermia could be observed already in late-stage spermatids in testis from GC-Δ8A mice (Fig. 5*I*).

### Swollen cytoplasm, disorganization of the mitochondrial sheath, and flagellar coiling in GC-Δ8A spermatozoa in epididymis

**Figure 6.**
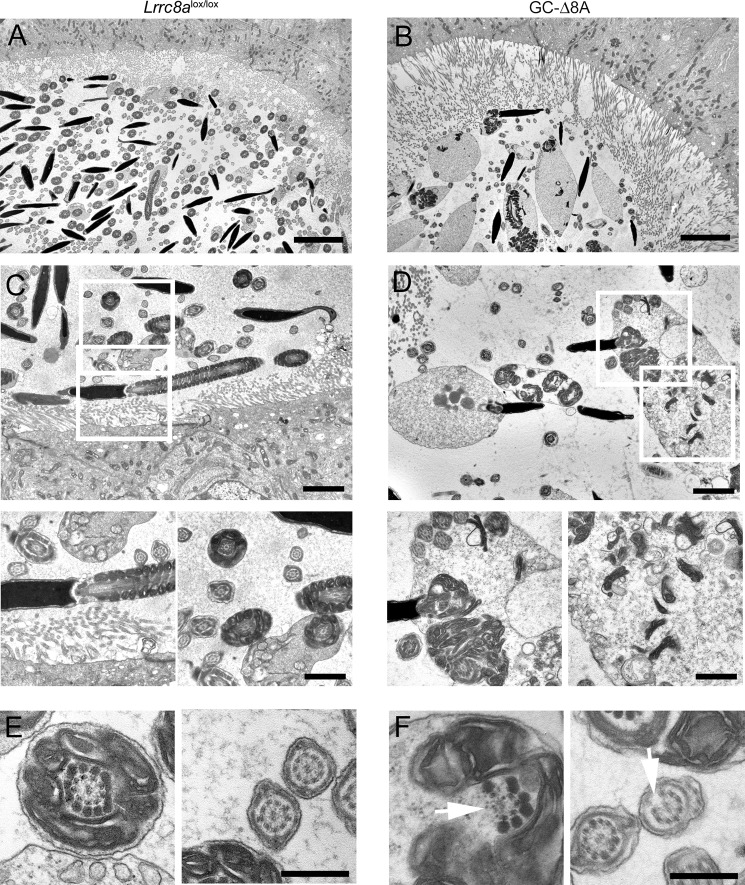
**Sperm in epididymis display profound morphological alterations.** *A* and *B*, TEM images of an overview cross-section from *Lrrc8a*^lox/lox^ control and GC-Δ8A mouse epididymis. Note the multiple swollen spermatozoa in the lumen of GC-Δ8A tubules. *Scale bars*, 5 μm (*A* and *B*). *C*, *Lrrc8a*^lox/lox^ control spermatozoa. Note the axoneme and mitochondrial sheath organization in *magnified regions* below. *D*, GC-Δ8A spermatozoa. Note the multiple cross-sections through the flagella without mitochondrial sheath, clustered mitochondria, and swollen cytoplasm. *Scale bars*, 2 μm (*C* and *D*). *E*, tail cross-sections of *Lrrc8a*^lox/lox^ control spermatozoa. *F*, cross-sections through flagellum of GC-Δ8A spermatozoon. Note axoneme abnormalities (*white arrows*). *Scale bars*, 500 nm (*E* and *F*).

In testes, the majority of GC-Δ8A spermatozoa appeared to have proper mitochondrial sheaths (Fig. 4, *C* and *E*) and also on EM level flagella displayed relatively normal midpiece regions (Fig. 5*M*). In contrast, morphological aberrations were much more prominent in epididymal sections of GC-Δ8A mice (Fig. 6, *A–D*). Control spermatozoa displayed well-developed heads, and their mitochondria were tightly organized around the axoneme in the midpiece region (Fig. 6, *A* and *C*), whereas GC-Δ8A spermatozoa had drastically enlarged cytoplasm and highly disorganized mitochondrial sheaths (Fig. 6, *B* and *D*). Mitochondria were disorganized and to a large extent detached from the axoneme, invading the cytoplasmic region (Fig. 6, *B* and *D*). Furthermore, the perinuclear cytoplasm often contained multiple cross-sections of flagella, indicating coiling of the tail (Fig. 6, *B* and *D*). In addition to malformed mitochondrial sheaths, some tail cross-sections also suggested a disorganization of the microtubules constituting the flagella core (Fig. 6*F*, control flagella for comparison Fig. 6*E*).

## Discussion

Given the complexity of spermatogenesis, it is not surprising that many genetic defects, which may affect several cell types, including hormone-producing cells, nurturing Sertoli cells and germ cells, may underlie male infertility. Several of the mutated genes encode ion channels and transporters that can affect fertility at various levels. For instance, mutations in components of sperm-specific CatSper Ca^2+^ channels entail infertility by impairing hyperactivated sperm motility without interfering with their development (42–45). Disruption of sperm-specific Slo3 K^+^ channels similarly impedes sperm activation and acrosome reaction (46), and sperm cell–specific disruption of the Golgi-resident Na^+^/H^+^ exchanger NHE8 impairs acrosome formation and leads to globozoospermia (36). Disruption of the widely expressed Orai1 store-operated Ca^2+^-channel interferes with the development of spermatozoa and entails tubular degeneration (47), whereas the infertility of mice lacking the TRPV6 cation channel was attributed to decreased Ca^2+^ reabsorption from the epididymal lumen (48). Less is known about roles of Cl^−^ channels in male fertility. Mutations in the CFTR Cl^−^ channel cause male infertility by a developmental defect, resulting in congenital absence of vas deferens (49), but *Cftr*^−/−^ mice are fertile (50), whereas loss-of-function mutations in ClC-2 result in another form of syndromal male infertility that prominently includes leukodystrophy and blindness as additional pathologies (33, 51, 52). Loss of ClC-2 is associated with severe degeneration of seminiferous tubules and a rapid, complete loss of male germ cells leading to Sertoli cell-only syndrome (33). It was attributed to a primary effect on Sertoli cells (33), but this hypothesis awaits confirmation by cell type–specific KOs.

Here we used Sertoli and germ cell–specific disruption of *Lrrc8a* to show that the male infertility observed in *Lrrc8a*^−/−^ mice and *ébouriffé* mice (28, 29), which express severely truncated LRRC8A proteins (30), is due to a loss of VRAC in germ cells. VRACs are heteromers of up to five different LRRC8 proteins, with LRRC8A being the only essential subunit (21). Hence, deletion of LRRC8A abolishes VRAC's transport of halide anions and of a plethora of organic compounds (21, 26, 27). Truncated LRRC8A mutants are largely stuck in the ER and are thus unable to carry the other LRRC8 subunits (LRRC8B–E) to the plasma membrane (21). The truncation of LRRC8A in *ébouriffé* mice thus results in drastically reduced, but not completely abolished, swelling-activated *I*_Cl,vol_ currents (30). This may explain that the pathology of *ébouriffé* mice is less severe than in *Lrrc8a*^−/−^ mice (28) which completely lack *I*_Cl,vol_. The germ cell phenotype of *ébouriffé* mice largely resembles that of GC-Δ8A mice but additionally includes abnormalities of sperm heads, which display defective acrosome formation, as well as abundant testes vacuolization (29). It seems counterintuitive that the more severe phenotype of *ébouriffé* mice may be caused by the less complete loss of VRAC transport activity compared with GC-Δ8A mice. However, although we could not detect abnormalities in Sertoli cell–specific LRRC8A KO mice, we cannot exclude the possibility that a simultaneous decrease of *I*_Cl,vol_ in both Sertoli and germ cells causes the more severe phenotype of *ébouriffé* mice.

The pathology of GC-Δ8A mice is most likely caused by a loss of VRAC-mediated transport of Cl^−^ or organic compounds. The prominent expression of LRRC8D in testis suggests that it may, in principle, transport organic compounds such as taurine, *myo*-inositol, and glutamate across the plasma membrane of germ cells, compounds that also serve as organic osmolytes (26). Intriguingly, the lumen of the epididymis displays high concentrations of organic osmolytes (53), which may be taken up by spermatozoa through VRACs and later serve as a reservoir of intracellular osmolytes. The notion that VRAC ablation impairs the RVD of germ cells was buttressed by the markedly increased cytoplasm of GC-Δ8A spermatozoa, by electron micrographs revealing reduced electron-dense cytoplasm and increased distance between ribosomes, and by tail angulation and coiling that was previously associated with impaired sperm volume regulation (7). Although these pathologies increased on the way from testis to epididymis, the first signs of cytoplasmic swelling were already observed with maturing spermatids in layer 2 of seminiferous tubules. Whereas spermatozoa experience (slow) changes in extracellular osmolarity on their way to, and within, the epididymis, it is assumed that they are not exposed to hypotonicity in seminiferous tubules (54). However, it cannot be excluded that ion transport processes of Sertoli cells create a hypotonic environment in the narrow clefts between them and germ cells, which would normally lead to only slight swelling of spermatozoa because the opening of VRAC would lead to an efflux of osmolytes and water. In this scenario, VRACs may play an important, although not exclusive, role in the drastic reduction of the cytoplasm of mature sperm. We propose that the other morphological changes, such as the disorganization of the mitochondrial sheath surrounding the axoneme and later the angulation and coiling of sperm tails, occur secondarily to the inability of spermatozoa to properly reduce their cytoplasm as observed in other KO mouse models (39, 41). These morphological changes appear sufficient to explain their reduced motility and the resulting infertility of KO mice (39, 41).

In conclusion, the volume-regulated anion channel VRAC is necessary, in a cell-autonomous manner, for the normal development of spermatozoa and hence male fertility. We suggest that the severe malformation of spermatids lacking VRAC is initiated by impaired cell volume regulation that results in swelling of the cytoplasm that impairs the elimination of excess cytoplasm as a prerequisite of further maturation. However, we cannot exclude that impaired transport of metabolites or signaling molecules across the plasma membrane of germ cells, enabled by the presence of LRRC8D, contributes to the pathology. *LRRC8A* might be considered as new candidate gene for human male infertility, probably always associated with several other symptoms such like those of the severely affected *Lrrc8a*^−/−^ and of *ébouriffé* mice. Although the latter mice display a somewhat milder phenotype, *Lrrc8a*+/− mice are fertile and appear also otherwise normal. It seems unlikely that an intermediate reduction of VRAC activity specifically causes male infertility without other symptoms, whereas a strong or complete loss of function may prevent patients from reaching puberty.

## Experimental procedures

### Mice

**Figure 7.**
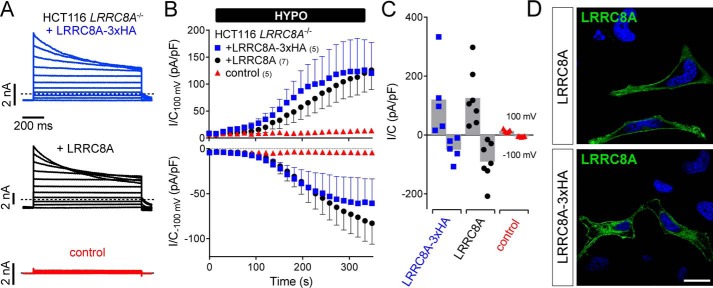
**Carboxyl-terminal HA epitopes do not alter functional properties of LRRC8A.** Whole-cell VRAC currents (*I*_Cl,vol_) measured in HCT116 cells in which *LRRC8A* was disrupted with CRISPR-Cas9 (21) and which were transfected with plasmids encoding WT LRRC8A (*black*), LRRC8A-3xHA (*blue*), or EGFP (as transfection control) (*red*). *A*, representative current traces of maximally activated *I*_Cl,vol_ in response to 2-s steps to voltages between −80 and 120 mV (Δ = 20 mV) from a conditioning potential of −80 mV. *Dashed lines* indicate zero current. *B*, time course of *I*_Cl,vol_ activation as reflected by current densities at −100 mV (*bottom*) and 100 mV (*top*) sampled every 15 s. *Error bars*, S.E. The number of cells is indicated in the legend. *C*, current densities of maximally activated I_Cl,vol_ at −100 and 100 mV. *D*, immunofluorescent labeling of LRRC8A in HeLa cells that were transfected with plasmids encoding LRRC8A and LRRC8A-3xHA, respectively. Note that both constructs can be detected at the plasma membrane. *Scale bar*, 20 μm.

Animal care and experiments were in accordance with the German animal protection laws and were approved by the Berlin authorities (LaGeSo). The generation of KI mice expressing β-gal driven by the endogenous *Lrrc8a* promoter and conditional *Lrrc8a*^lox/lox^ mice using targeted ES cells obtained from EUCOMM (European Conditional Mouse Mutagenesis Program; *Lrrc8a*^tm2a^ (EUCOMM) Hmgu) have been described elsewhere (32). KI mice expressing an LRRC8A-3xHA fusion protein from the endogenous *Lrrc8a* promoter were generated by CRISPR-Cas9 mediated recombination in zygotes that were implanted into pseudo-pregnant NMRI female mice by the transgenic core facility of the Max-Delbrück-Centrum für Molekulare Medizin (Berlin, Germany). Zygotes were injected with sgRNA, targeting vector, and Cas9 mRNA and protein into one pronucleus following standard procedures (55). To obtain the zygotes for microinjection, *Lrrc8a*^lox/lox^ males were mated with super-ovulated C57BL/6N females (Charles River, Sulzbach, Germany), resulting in *Lrrc8a*^HA/HA^ and *Lrrc8a*^lox-HA/lox-HA^mice, respectively. Sequence encoding for the triple human influenza HA (5′-tacccatacgatgttccagattacgctggctatccctatgacgtcccggactatgcaggatcctatccatatgacgttccagattacgctgtt-3′) tag was introduced at the carboxyl terminus of LRRC8A. Initial control experiments showed that the tag changed neither properties of I_Cl,vol_ (Fig. 7, *A–C*) nor the localization of LRRC8A in transfected cells (Fig. 7*D*). For Sertoli cell–specific and male premeiotic germ cell–specific deletion of *Lrrc8a*, respectively, *Lrrc8a*^lox/lox^ or *Lrrc8a*^lox-HA/lox-HA^ mice were crossed to *AMH*-Cre mice (B6-Tg(Amh-cre)8815Reb/J (34)), which express the Cre- recombinase under the Sertoli cell-specific *Anti-Müllerian hormone* promoter, or *Stra8*-iCre mice (B6.FVB-Tg(Stra8-cre)1Reb/LguJ (35)), which express the Cre-recombinase under the germ cell–specific *Stimulated by retinoic acid 8* promoter, respectively. These mice were obtained from the National Institute for Agronomic Research and The Jackson Laboratory (stock 017490), respectively. Additionally, we crossed *Lrrc8a*^lox/lox^ mice with *Ngn3*-Cre mice that also express the recombinase in male germ cells (B6.FVB(Cg)-Tg(Neurog3-cre)C1Able/J; Jackson stock 006333 (56)) and obtained results similar to those observed upon *Stra8*-iCre mediated *Lrrc8a* disruption (*data not shown*). Green sperm mice (40) were obtained from RIKEN (B6D2-Tg(CAG/Su9-DsRed2, Acr-EGFP) RBGS002Osb, stock RBRC03743) and crossed with the germ cell–specific *Lrrc8a*^−/−^ mice.

### Antibodies

Polyclonal antibodies against the individual LRRC8 subunits had been raised in rabbits (Pineda-Antikörper-Service, Berlin, Germany), with their specificity being ascertained by Western blotting using KO cell lines as controls as described (21, 26, 27). LRRC8A was also detected as HA-tagged fusion protein, using a monoclonal rabbit anti-HA tag antibody (Cell Signaling, catalog no. 3724, 1:1000). Other antibodies used were: chicken anti-GFP (Aves labs, GFP-1020, 1:1000), mouse anti-dsRed (Clontech, 632393, 1:500), mouse anti-α-tubulin (Sigma, T6199, 1:1000). Peanut agglutinin (PNA) coupled to Alexa fluorophore 568 was used as acrosomal marker (Molecular Probes, L32458, 1:800). For the detection of EGFP-tagged acrosin and DsRed2-tagged mitochondria on testis sections of green sperm mice (40), GFP booster coupled to the dye Atto488 (Chromotek, gba488, 1:200) and RFP booster coupled to the dye Atto647N (Chromotek, rba488, 1:200) were used, respectively. Secondary antibodies coupled to different Alexa fluorophores (488, 555, or 647; 1:1000) were from Molecular Probes, and antibodies coupled to horseradish peroxidase were from Jackson ImmunoResearch (1: 10 000). DAPI was obtained from Invitrogen (1:1000).

### Immunocytochemistry of HeLa cells

HeLa cells were maintained in DMEM, supplemented with 10% FCS and 1% penicillin/streptomycin (all from PAN Biotech) at 37 °C and 5% CO_2_. For immunocytochemistry, the cells were seeded on poly-l-lysine (Sigma)–coated coverslips and transiently transfected with plasmids encoding either untagged LRRC8A or LRRC8A-3xHA using FuGENE 6® transfection reagent (Promega). 48 h post-transfection, the cells were fixed in ice-cold MeOH at −20 °C for 15 min. Following a blocking step in 3% BSA, 0.1% saponin/PBS, the cells were sequentially incubated with anti-LRRC8A antibody and secondary antibody coupled to Alexa fluorophore 488/DAPI for 1 h each in blocking solution. Images were acquired on Zeiss LSM 510 META laser scanning microscope. Image processing was done with the ZEN software (Zeiss) and Adobe Photoshop.

### Isolation and immunocytochemistry of mouse spermatozoa

The mice were killed by cervical dislocation, and cauda epididymides were dissected immediately. They were thoroughly cleaned from fat, transferred to HEPES-buffered saline containing 135 mm NaCl, 5 mm KCl, 2 mm CaCl_2_, 1 mm MgSO_4_, 20 mm HEPES, 5 mm glucose, 10 mm lactic acid, 1 mm sodium pyruvate, pH 7.4, with NaOH (320 mosM) (45) and ruptured. The sperm were allowed to move out for 5 min at 37 °C. To visualize mitochondrial sheaths, the spermatozoa were incubated with MitoTracker® green (Molecular Probes) at a final concentration of 100 nm for 15 min at 37 °C, centrifuged 1 min at 10,000 × *g*, and resuspended in PBS. The sperm were smeared on coverslips and air-dried overnight, fixed with 1–4% PFA, incubated in 30 mm glycine/PBS for 10 min, and permeabilized for 4 min with 0.2% Triton X-100 in 3% BSA/PBS. The cells were incubated with PNA coupled to Alexa fluorophore 568 to detect the acrosomal cap and DAPI to reveal nuclei diluted in 3% BSA/PBS supplemented with 0.1% Triton X-100 for 1 h at room temperature protected from light. To detect acrosomal caps and mitochondrial sheaths of spermatozoa isolated from green sperm mice, incubation with MitoTracker® green was omitted. After permeabilization, spermatozoa were incubated with 3% BSA, 0.1% Triton X-100, PBS containing anti-GFP and anti-DsRed antibodies for 4–16 h at 4 °C and for 1 h with respective secondary antibodies coupled to Alexa fluorophores and DAPI in the dark at room temperature. Images were acquired on Zeiss LSM 880 laser scanning confocal microscope using the ZEN software (Zeiss). Image processing was done with the ZEN software (Zeiss) and Adobe Photoshop.

### Measurement of sperm motility

Isolated spermatozoa were incubated in human tubular fluid medium, containing 102 mm NaCl, 4.7 mm KCl, 2 mm CaCl_2_, 0.2 mm MgCl_2_, 0.37 mm KH_2_PO_4_, 2.78 mm glucose, 18.3 mm lactic acid, 0.33 mm sodium pyruvate, 25 mm HCO_3_^−^, and 4 mg ml^−1^ BSA (275 mosM) (57) for 5 min at 37 °C. Sperm motility was assessed using the IVOS sperm analyzer version 12 (Hamilton Thorne Research, Beverly, MA) using parameters described previously (36).

### Histology and immunohistochemistry

For histological analysis, deeply anesthetized mice were perfused with 4% PFA in PBS and collected organs were postfixed in the same solution overnight at 4 °C. Paraffin sections of 6-μm thickness (Cool-cut Microm HM 355 S, Thermo Scientific) were stained with hematoxylin and eosin (H&E) following standard protocols. For X-Gal staining, organs were transferred to 30% sucrose in PBS after 2 h of postfixation. After 24–48 h, organs were embedded in Tissue-Tek® O.C.T.^TM^ compound (Sakura) and sliced using a Cool-Cut microtome (Microm HM 560, Cryostat-Series, Thermo Scientific) to obtain 6-μm frozen sections. Staining was performed as previously described (36). Images of H&E and X-Gal stainings were taken with an AxioCam MRa5 (Zeiss) on an Axiophot microscope (Zeiss) using the ZEN software (Zeiss). All histological analyses were reproduced in at least three independent experiments. For immunohistochemistry, anesthetized mice were perfused with 1% PFA in PBS, and the collected organs were incubated in 30% sucrose in PBS without postfixation. For immunofluorescent staining of organs from LRRC8A-3xHA KI mice, frozen sections were postfixed with 1% PFA/PBS, incubated in 30 mm glycine/PBS, blocked in 5% normal goat serum in PBS, supplemented with 0.25% Triton X-100, and incubated with primary anti-HA tag antibody in 1% BSA/PBS supplemented with 0.25% Triton X-100 overnight at 4 °C. Secondary antibody was coupled to Alexa fluorophore 488 and counterstained with DAPI for 60 min at room temperature in the dark. Immunohistochemistry of epididymides from green sperm mice was performed on frozen sections as immunocytochemistry of spermatozoa. Immunohistochemistry of testes from green sperm mice was performed on paraffin sections with GFP and RFP boosters coupled to Atto fluorophores (Chromotek). The sections were permeabilized with 0.5% Triton X-100/PBS, blocked in 3% BSA/PBS and incubated with boosters and DAPI diluted in blocking buffer for 1 h at room temperature. Images were acquired on Zeiss LSM 880 laser scanning confocal microscope using the ZEN software (Zeiss).

### Transmission EM

Following transcardial perfusion of deeply anesthetized mice with 4% PFA, 2% glutaraldehyde in PBS, testes and epididymides were collected and postfixed overnight in the same solution. After washing in cacodylate buffer, tissue was osmificated in 1% osmium tetroxide and 1.5% potassium cyanoferrat (III) in water followed by washing in 1% aqueous uranyl acetate. After dehydration in methanol gradients, tissue was infiltrated by epoxy resin with the help of propylene oxide and embedded in pure epoxy resin. Following polymerization, tissue was trimmed and sectioned. Ultrathin sections were imaged at a Zeiss 900 transmission electron microscope equipped by Morada G2 digital camera. Tissues from three control and four *knock-out* animals were extensively analyzed.

### Western blotting analyses

To obtain membrane fractions from mouse tissue, organs were homogenized in 20 mm Tris-HCl, pH 7.4, 140 mm NaCl, 2 mm EDTA with protease inhibitors (4 mm Pefabloc®, Complete EDTA-free protease inhibitor mixture, Roche) using an IKA T 10 basic ULTRA-TURRAX® disperser. Tissue homogenate was cleared by centrifugation for 10 min at 1000 × *g* twice, and membrane fractions were pelleted from the cleared homogenate by ultracentrifugation for 30 min at 100,000 × *g*. The membrane pellet was resuspended by sonication in 50 mm Tris-HCl, pH 6.8, 140 mm NaCl, 0.5 mm EDTA, 1% SDS (w/v), 1% Triton X-100 (w/v) with protease inhibitors. Equal amounts of protein (30 μg/lane) were separated via SDS-PAGE and blotted onto nitrocellulose. Western blots were probed with the indicated antibodies. Tubulin served as loading control.

### Electrophysiology

*LRRC8A*^−/−^ HCT116 cells (21) were maintained in McCoy's 5A medium supplemented with 10% FBS and 1% penicillin/streptomycin (all from PAN Biotech) at 37 °C and 5% CO_2_. For experiments, the cells were plated onto gelatin-coated coverslips and transfected using the Lipofectamine 2000 (Life Technologies, Inc.) transfection reagent. pEGFP-N1 was co-transfected (1:10) for visual identification of cells.

The whole-cell patch clamp technique was used to measure *I*_Cl,vol_ as described (21, 58). The pipette solution contained 40 mm CsCl, 100 mm cesium methanesulfonate, 1 mm MgCl_2_, 1.9 mm CaCl_2_, 5 mm EGTA, 4 mm Na_2_ATP, and 10 mm HEPES, pH 7.2, with CsOH (290 mOsm). The hypotonic saline used to elicit *I*_Cl,vol_ contained 105 mm NaCl, 6 mm CsCl, 1 mm MgCl_2_, 1.5 mm CaCl_2_, 10 mm glucose, 10 mm HEPES, pH 7.4, with NaOH (240 mOsm). The currents were recorded with an EPC-10 USB patch clamp amplifier and PatchMaster software (HEKA Elektronik). The cells were held at −30 mV and a 2.6-s ramp protocol from −100 to 100 mV was applied every 15 s to monitor the time course of *I*_Cl,vol_ current densities. Maximally activated *I*_Cl,vol_ was further characterized by 2-s step protocols from −80 to 120 mV in 20-mV increments, preceded and followed by 0.5-s steps to −80 mV to ensure full recovery from inactivation.

## Author contributions

J. C. L. and T. J. J. conceptualization; J. C. L., D. P., and F. U. data curation; J. C. L., D. P., and F. U. formal analysis; J. C. L., D. P., F. U., and T. J. J. validation; J. C. L., D. P., and F. U. investigation; J. C. L., D. P., and F. U. visualization; J. C. L., D. P., and F. U. methodology; J. C. L., D. P., and T. J. J. writing-original draft; J. C. L., D. P., F. U., and T. J. J. writing-review and editing; T. J. J. resources; T. J. J. software; T. J. J. supervision; T. J. J. funding acquisition; T. J. J. project administration.

## Footnotes

2 The abbreviations used are:

RVD: regulatory volume decrease
VRAC: volume-regulated anion channel
KI: *knock-in*
KO: *knock-out*
HA: hemagglutinin
EGFP: enhanced green fluorescent protein
TEM: transmission EM
PNA: peanut agglutinin
DAPI: 4′,6′-diamidino-2-phenylindole
H&E: hematoxylin and eosin.
